# Supplementary material for: Diagnostic Performance and Tolerability of Saliva and Nasopharyngeal Swab Specimens in the Detection of SARS-CoV-2 by RT-PCR
Source: Microbiol Spectr. 2023 Apr 24;11(3):e05324-22. doi: 10.1128/spectrum.05324-22 (PMC10269602; doi:10.1128/spectrum.05324-22)
Supplement: Supplemental file 1 — Table S1. Download spectrum.05324-22-s0001.pdf, PDF file, 0.6 MB [file spectrum.05324-22-s0001.pdf]

Supplementary Appendix

Diagnostic Performance and Tolerability of Saliva and Nasopharyngeal Swab Specimens in Detection of SARS-CoV-2 by RT-PCR

Jaakko Ahti, Riikka Österback, Anniina Keskitalo, Kati Mokkala, Siina Vidbäck, Ville Veikkolainen,

Tytti Vuorinen, Ville Peltola, Antti J. Hakanen, Matti Waris, Miia Laine

TABLE S1. Cycle Threshold (C_T) values[a] of participants with discordant results between Nasopharyngeal swab (NPS) and Saliva specimens.

LDT E gene NPS/Saliva	PerkinElmer N gene NPS/Saliva	PerkinElmer ORF1ab gene NPS/Saliva	PerkinElmer Plus N/E gene NPS/Saliva	PerkinElmer Plus ORF1ab gene NPS/Saliva
36.7/40.0	34.2/40.0	34.7/40.0	33.5/40.0	33.7/40.0
40.0/40.0	40.0/36.8	40.0/36.7	40.0/40.0	40.0/40.0
40.0/40.0	40.0/40.0	39.1/40.0	40.0/40.0	37.8/40.0
30.5/40.0	28.1/40.0	30.3/40.0	29.0/40.0	29.5/40.0
40.0/28.0	40.0/29.5	40.0/27.6	40.0/28.0	40.0/26.7
40.0/27.7	36.3/32.8	37.7/40.0	36.8/25.8	35.5/24.7
33.2/40.0	28.1/40.0	30.8/40.0	28.1/38.3	29.2/37.6
34.2/40.0	31.5/38.0	33.2/37.4	31.7/36.9	32.5/36.3
40.0/40.0	40.0/40.0	40.0/40.0	37.8/40.0	40.0/40.0
40.0/31.8	40.0/34.8	40.0/31.8	40.0/32.0	40.0/31.0
40.0/37.5	38.3/37.3	39.1/38.0	39.1/36.7	39.7/36.1
40.0/40.0	39.9/40.0	38.3/40.0	40.0/40.0	40.0/40.0
40.0/40.0	40.0/40.0	40.0/40.0	37.7/40.0	39.7/40.0
40.0/28.6	38.4/30.3	36.8/28.5	36.6/29.4	37.1/28.3
35.4/40.0	33.5/40.0	32.4/40.0	32.6/40.0	31.7/40.0
27.0/40.0	24.7/40.0	25.0/40.0	22.4/40.0	22.6/40.0
40.0/40.0	40.0/40.0	39.1/40.0	38.4/40.0	37.0/40.0

[a] C_T value of 40.0 was given for all negative results presented in this table.

LDT = Laboratory-developed test
